# Global Phosphoproteomic Analysis Reveals the Involvement of Phosphorylation in Aflatoxins Biosynthesis in the Pathogenic Fungus *Aspergillus flavus*

**DOI:** 10.1038/srep34078

**Published:** 2016-09-26

**Authors:** Silin Ren, Mingkun Yang, Yu Li, Feng Zhang, Zhuo Chen, Jia Zhang, Guang Yang, Yuewei Yue, Siting Li, Feng Ge, Shihua Wang

**Affiliations:** 1 Key Laboratory of Pathogenic Fungi and Mycotoxins of Fujian Province and School of Life Sciences, Fujian Agriculture and Forestry University, Fuzhou, 350002, China; 2 Key Laboratory of Algal Biology, Institute of Hydrobiology, Chinese Academy of Sciences, Wuhan, 430072, China

## Abstract

*Aspergillus flavus* is a pathogenic fungus that produces toxic and carcinogenic aflatoxins and is the causative agent of aflatoxicosis. A growing body of evidence indicates that reversible phosphorylation plays important roles in regulating diverse functions in this pathogen. However, only a few phosphoproteins of this fungus have been identified, which hampers our understanding of the roles of phosphorylation in *A. flavus*. So we performed a global and site-specific phosphoproteomic analysis of *A. flavus*. A total of 598 high-confidence phosphorylation sites were identified in 283 phosphoproteins. The identified phosphoproteins were involved in various biological processes, including signal transduction and aflatoxins biosynthesis. Five identified phosphoproteins associated with MAPK signal transduction and aflatoxins biosynthesis were validated by immunoblotting using phospho-specific antibodies. Further functional studies revealed that phosphorylation of the MAP kinase kinase kinase Ste11 affected aflatoxins biosynthesis in *A. flavus*. Our data represent the results of the first global survey of protein phosphorylation in *A. flavus* and reveal previously unappreciated roles for phosphorylation in the regulation of aflatoxins production. The generated dataset can serve as an important resource for the functional analysis of protein phosphorylation in *A. flavus* and facilitate the elucidation of phosphorylated signaling networks in this pathogen.

*Aspergillus flavus* is associated with disease in plants, animals and humans and is the common causal agent of aflatoxins contamination. Aflatoxins are polyketide-derived secondary metabolites that are produced by both *A. flavus* and *A. parasiticus*, which are highly toxic, mutagenic, and carcinogenic to animals and humans^1^. Food contaminated by aflatoxins may result in acute aflatoxicosis and even death. Chronic exposure to sublethal concentrations of aflatoxins leads to hepatocellular carcinoma, growth impairment, and immunological suppression^2^. To counter the increasing threat of aflatoxicosis, it is essential to understand the molecular mechanisms underlying biosynthesis of aflatoxins and to identify new drug targets for the design of novel therapeutic agents.

Reversible protein phosphorylation at serine (S), threonine (T), and tyrosine (Y) residues is the most widespread type of post-translational modification (PTM) in cells, regulating crucial cellular functions, including signal transduction, cell proliferation, and development^3^. Phosphorylation is catalyzed by specific protein kinases and phosphatases; in doing so, phosphorylation-based signaling is regulated by the antagonizing catalytic activities of protein kinases and protein phosphatases in all eukaryotic cells^4^,^5^. In fact, almost all cell signaling processes are dependent on protein phosphorylation and dephosphorylation. Over the past several years, accumulated experimental evidence has demonstrated that protein kinases can regulate diverse cellular programs, based on cues derived from the cell surface, the metabolic state of the cell, and the environment of the cell, and play a central role in signaling pathways^6^. Moreover, protein kinases and phosphatases, as well as their potential substrates, have now become major drug targets for a wide variety of diseases, highlighting that understanding of phosphorylation-based signaling pathways can be expected to provide new insights into the design of novel therapeutic agents for diseases^7^. Recent advances in phosphopeptide enrichment, followed by liquid chromatography-tandem mass spectrometry (LC-MS/MS), have made it possible to study proteins that are regulated by phosphorylation at system level, and a large number of phosphorylation events have been identified in many model organisms, including *Saccharomyces cerevisiae, Synechococcus* spp., *Drosophila melanogaster*, mouse, and human^8^,^9^,^10^,^11^,^12^. Moreover, global analysis of the phosphoproteome has also been reported for pathogenic fungi, including *A. nidulans* and *Candida albicans*^13^,^14^. These studies presented an inventory of phosphoproteins and revealed the diverse functions of phosphorylation in the cell cycle, apoptosis, metabolism, signal transduction, proliferation and development.

In recent years, major advances have been made in elucidating the mechanism underlying aflatoxins production. For example, dephosphorylation of calcineurin-mediated enzymes ensures production of aflatoxins in the pathogenic fungus *A. parasiticus* NRRL 2999^15^. The transcription factor AflR can be negatively regulated by phosphorylation and the expression of aflatoxins biosynthetic genes (except *estA*) is regulated by *aflR*^1^,^16^. Additionally, most likely, turnover of AflR is mediated by a phosphorylation cascade, reflecting the requirements for a rapid response to external metabolic signals^17^. No information is available regarding kinase substrate specificity in *A. flavus* NRRL 3357, despite the fact that, according to genome analysis, this pathogenic fungus contains at least 119 kinases and 54 phosphatases. Global analysis of phosphorylated proteins in this pathogen would facilitate understanding of the role of phosphorylation in aflatoxins biosynthesis and signal transduction pathways, and this analysis could provide a firm basis for future research. However, to our knowledge, only a few *A. flavus* phosphoproteins have been identified to date, and little progress has been made in ascertaining precisely which *A. flavus* proteins are phosphorylated, which kinases/phosphatases are involved in this process, and which cellular functions are targeted by this important PTM.

To fill this gap, we performed a global phosphoproteomic analysis of this pathogen, using a combination of TiO_2_ enrichment and LC-MS/MS analyses. In total, we identified 598 phosphorylation sites on a total of 283 phosphoproteins from *A. flavus*. Consistent with previous reports, unbiased bioinformatics analyses indicated that a large proportion of these phosphorylation sites are present on proteins involved in signal transduction pathways. Furthermore, we also identified phosphorylated proteins involved in metabolic pathways, related to aflatoxins biosynthesis. Immunoblotting analysis indicated that phosphorylation could play a key role in responses to environmental stimuli and aflatoxins generation. Further functional studies revealed that phosphorylation of Ste11 affected colonial morphology, conidial generation, sclerotial formation and aflatoxins biosynthesis in *A. flavus*. These results provide novel insights into the range of functions regulated by phosphorylation in *A. flavus*. The findings of this study may help improve the general understanding of the mechanism underlying aflatoxins biosynthesis and may lead to the development of innovative strategies to control aflatoxins production.

## Results

### Establishment of *A. flavus* phosphoproteome

**Figure 1:**
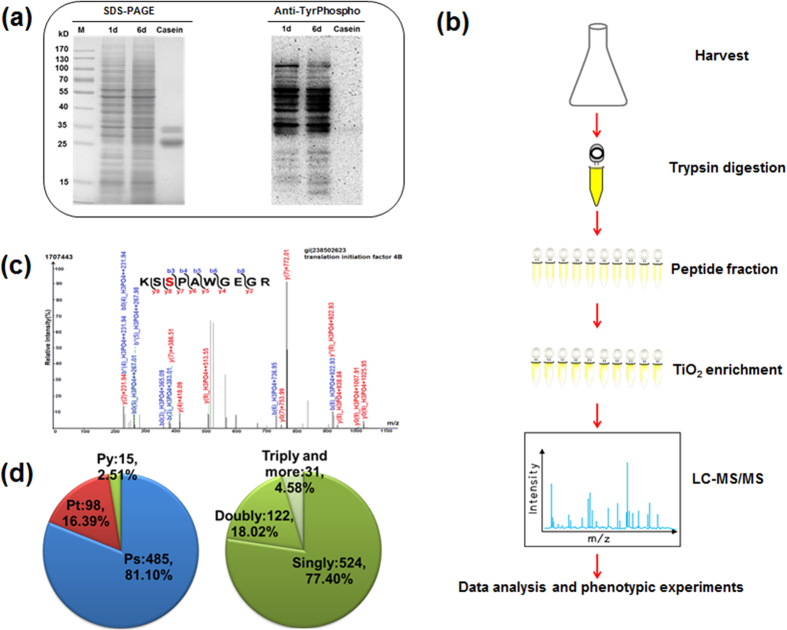
Overview of the experimental design and workflow. (**a**) Verification of phosphoproteins present in *Aspergillus flavus* NRRL 3357. The SDS-PAGE gel was stained with Coomassie Brilliant Blue or transferred to a PVDF membrane and incubated with a polyclonal anti-phospho-Tyr antibody. 1 d, protein obtained from *A. flavus* cultured for 1 d; 6 d, protein obtained from *A. flavus* cultured for 6 d. Casein was used as a positive control for Tyr phosphorylation. (**b**) Experimental scheme for this study. Proteins were harvested via filtration and centrifugation, followed by trypsin digestion, prefractionation and TiO_2_ enrichment of phosphopeptides. Two types of mass spectrometers were used to analyze enriched phosphopeptides. (**c**) A representative MS/MS spectrum. The peptide (KSSPAWGEGR) is from translation initiation factor 4B, and the phosphorylated Ser site (red) is indicated. (**d**) The distribution of phospho-Ser (pS), phospho-Thr (pT), and phospho-Tyr (pY) residues and the distribution of peptides with single, double, triple, or more phosphorylation sites are shown.

In order to gain an overview of the diversity and relative abundance of phosphoproteins from different physiological/developmental stages of *A. flavus*, western blotting analysis was performed using a polyclonal anti-phosphotyrosine antibody. As shown in Fig. 1a, the global tyrosine-phosphorylation level varied when the cells were grown in different growth stages (1 d or 6 d). To our knowledge, the production of aflatoxins and toxin levels increase at maximum rates during a transition from the exponential growth phase to the stationary phase^18^. Our TLC analysis showed that aflatoxins were not produced when *A. flavus* was cultivated for only 1 d, whereas high levels of aflatoxins were generated after cultivation for 6 d (Supplementary Fig. S1). These observations suggested that phosphorylation was likely to link with the aflatoxins biosynthesis in *A. flavus*.

To identify the phosphorylation sites and phosphoproteins in *A. flavus*, an MS-based high-throughput proteomic approach was used (Fig. 1b). In total, 598 phosphorylation sites in 283 phosphoproteins were identified with an estimated FDR of less than 1%. Details of the phosphopeptides identified, including the scores obtained with the search algorithm and the PTM scores, are provided in Supplementary Table S1. All the raw data files and the annotated spectra of all phosphopeptides were submitted to the publicly accessible database PeptideAtlas (dataset ID PASS00400). Figure 1c shows a representative analysis of a phosphopeptide sequence from a translation initiation factor and assignment of the phosphorylation site. Moreover, we calculated the number of each modification site (phosphoserine [pS], phosphothreonine [pT] and phosphotyrosine [pY]) in the phosphoproteome of *A. flavus* in order to assess the distribution of phosphorylation sites. In agreement with other eukaryotic cells, the 598 phosphorylation sites, pS was most strongly represented (81.1%), followed by pT (16.4%), and pY (2.5%) (Fig. 1d). We reasoned that multiple phosphorylation sites on one protein may play an important role in fine-tuning of regulatory functions in *A. flavus*^19^. We also compared the *A. flavus* phosphoproteome to those of the fungi *A. nidulans* and *S. cerevisiae*, reported by other groups^11^,^13^. Interestingly, our findings also revealed that the phosphorylated proteins identified were likely to be evolutionarily conserved in fungal cells (Supplementary Table S2).

### Functional Classification and Enrichment of Phosphorylated Proteins

**Figure 2:**
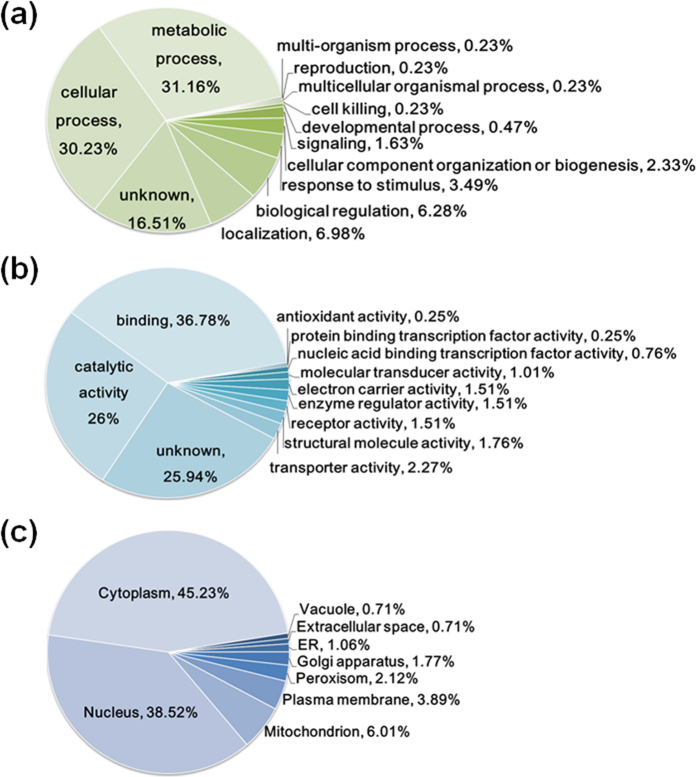
Pie charts showing the distribution of all identified phosphorylated proteins, as categorized according to biological processes (**a**), molecular functions (**b**), and cellular locations (**c**). The results were based on information provided by data analyses with Blast2GO and YLoc.

To better understand phosphorylation in *A. flavus*, we performed the Gene Ontology (GO) functional annotation analysis of all identified phosphoproteins according to the biological process, molecular function and cellular compartment. Our results showed that the phosphoproteins were distributed throughout a variety of biological processes, including metabolic processes, cellular processes, and biological regulation (Fig. 2a and Supplementary Table S3). Owing to the broad importance of phosphoproteins in signal transduction, it was not surprising that some phosphoproteins were annotated in the categories of stimulus and signaling, inferring that phosphorylation may play a vital role in the stress response. From the molecular functional perspective, the majority of the proposed functions entailed binding to targets and enzymatic activity (Fig. 2b and Supplementary Table S3). To date, the specific functions of 25.94% of identified phosphoproteins have not been reported. Among the 283 phosphorylated proteins of *A. flavus* NRRL 3357, according to the subcellular localization prediction program YLoc, 45.23% were categorized in the “cytoplasm” GO category and 38.52% in the “nucleus” (Fig. 2c and Supplementary Table S4). Additionally, we also found that phosphoproteins were associated with the mitochondrion (6.01%), plasma membrane (3.89%), peroxisome (2.12%) and other cellular components.

To gain further insight into the potential functional implications of phosphorylation, we carried out GO term enrichment using DAVID tool (Supplementary Fig. S2 and Supplementary Table S5). The majority of the identified phosphoproteins were mostly enriched in transport, such as intracellular transport (*p* = 9.67E-04), nucleocytoplasmic transport (*p* = 7.85E-03) and so on, suggesting that protein phosphorylation may play a role in the regulation of protein transport, as previously described^20^. Consistently, GO enrichment analysis of molecular function further illustrated that the phosphoproteins were related closely to the binding of various targets and enzymatic activity. Notably, since many proteins were assigned to protein kinase activity (*p* = 7.94E-03, *p* = 1.49E-03) and phosphotransferase activity (*p* = 1.32E-02) within the enzymatic activity cluster, it was easy to associate that phosphoproteins may play a role in signal transduction. Furthermore, phosphoproteins were enriched in the nuclear pore (*p* = 1.36E-02), pore complex (*p* = 1.36E-02) and nuclear envelope (*p* = 1.59E-02) in the cellular component category. Taken together, these results implied that the identified phosphoproteins had a wide distribution of cellular localizations and functions, and phosphorylation may play an important role in the regulation of diverse cellular processes in *A. flavus*.

### Phosphorylation Motifs and Potential Kinases Targeting Identified Phosphorylation Sites

**Figure 3:**
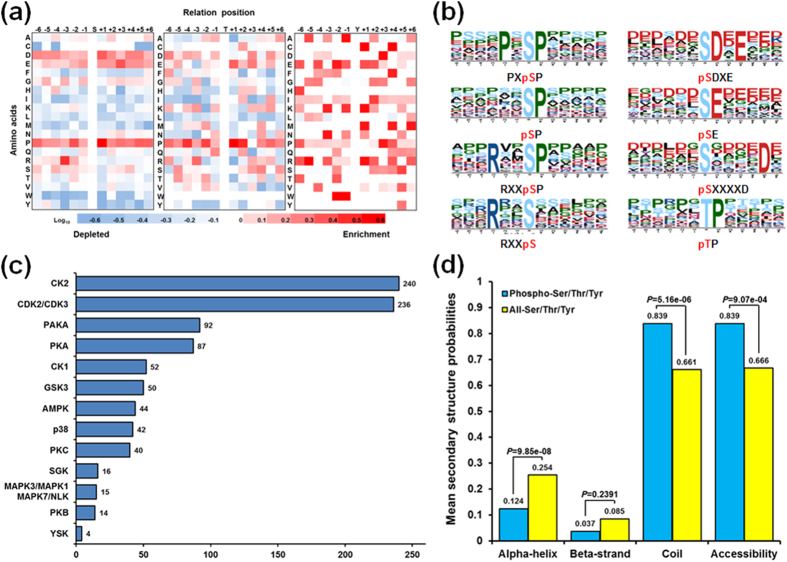
Bioinformatics of phosphorylation sites. (**a**) Intensity map showing the relative abundance of 12 amino acid residues flanking the class one phosphorylation sites. Different colors in the intensity map represent log_10_ of the ratio of amino acid frequency occurring within 13 residues from the pS/pT/pY residue versus within 13 amino acids from all S/T/Y residues. (**b**) *In vivo* phosphorylation sites, determined by Motif-X. The height of each amino acid represents the frequency of this residue occurring at the position of pS/pT. (**c**) Bar chart showing kinases targeting the class one phosphorylation sites, determined with NetworKIN. Only the results with both a NetworKIN score ≥1.0 and a String score ≥0.6 were considered. (**d**) Predicted secondary structures and accessibility of phosphorylation sites. Probabilities of different secondary structures (alpha-helix, beta-strand and coil) and accessibility of pS/pT/pY were compared with those of all S/T/Y residues in all phosphoproteins identified in this study.

The large-scale phosphoproteome enabled us to analyze site-specific phosphorylation motifs. We further compared the occurrences of each amino acid (six amino acids upstream and downstream of the phosphorylation site) with the entire set extracted from the *A. flavus* proteome (Fig. 3a). Interestingly, we observed a strong preference for proline (P) in both pS- and pT-containing peptides, reminiscent of classical proline-directed motifs. The residue preference for pS-containing peptides was for P at −2 and +1 positions relative to the pS site, while the presence of this amino acid at the +1 position was also highly favored for pT-containing peptides. Furthermore, aspartate (D) at the +1 position and glutamate (E) at the +3 position appeared to be slightly enriched in pS-containing peptides. We further validated and refined the intensity map data by examining significant phosphorylation motifs using the motif-identification algorithm Motif-X. As expected from the position-specific intensity maps, we identified four classical P-directed phosphorylation motifs, which were common among the most prevalent phosphorylation motifs (Fig. 3b), such as [PXpSP], [RXXpSP], [pSP], and [pTP]. In addition, four other motifs were found, including three acidic motifs, [pSDXE], [pSE], and [pSXXXXD], which were recognized as the substrates for casein kinase II and G protein-coupled receptor kinase 1^21^,^22^, and one basophilic motif, *viz.* [RXXpS], which was a specific recognition site for PKA^23^.

Previous studies have demonstrated that protein phosphorylation catalyzed by eukaryotic protein kinases (ePKs) is the most intensively studied PTM in eukaryotic cells and ePKs belong to one of the largest protein families^24^. The availability of the *A. flavus* phosphoproteome allowed us to infer kinase–substrate relationships in this fungus and to explore the extent of their phosphorylation pathways. Possible kinase groups targeting each of the 598 phosphorylation sites were predicted by NetworKIN (version 2) (Fig. 3c and Supplementary Table S6). After applying NetworKIN filtering criteria (String score ≥0.6 and NetworKIN score ≥1.0), 932 pairs of kinase–substrate relationships were predicted, comprising 13 serine/threonine kinase families for all identified phosphorylation sites. The most frequent serine/threonine kinase families were CK2 (240), CDK2/3 (236), PAKA (92), and PKA (87), which was consistent with the distribution of enriched motifs in the phosphorylation motif analysis. For example, we discovered three representative acidic motifs that were established as CK2 substrate recognition motifs. In addition, the CDK2/CDK3 group of kinases was comprised of basophilic serine/threonine kinases that can recognize specific motifs containing a conserved P immediately after a pS residue^25^, revealing that the detected motif [RXXpSP] could be recognized by CDK kinase. In this study, we found that the PKA group specifically recognized one basophilic motif, [RXXpS]. The other frequently observed phosphoserine/threonine kinase was p38, whose substrate recognition motif was a P-directed motif. The p38 MAPK family members are usually activated by cellular stress, including UV irradiation, heat shock, high osmotic stress, lipopolysaccharide, protein synthesis inhibitors and proinflammatory cytokines. Moreover, we identified several protein kinases belonging to the MAPK1/MAPK3/MAPK7/NLK group, which represented the best characterized MAPK signaling pathway.

### Local Structural Properties of Phosphorylation Sites

We next predicted the local secondary structures and solvent accessibilities of pS/pT/pY and all S/T/Y of *A. flavus* phosphoproteins using NetSurfP^26^. The pS/pT/pY showed a significantly different preference for secondary structure as compared with all S/T/Y (Fig. 3d). In accordance with previous report^27^, pS/pT/pY were found more frequently in unstructured coiled regions (83.9%, *p* = 5.161E-06) and less frequently in ordered helical regions (12.4%, *p* = 9.853E-08) than all S/T/Y, which is quite different from those of lysine PTM events^28^. In addition, the phosphorylation sites for solvent accessibility were further evaluated and the result showed that an average relative side-chain solvent accessibility of 83.9, which was significantly higher than that of all S/T/Y (66.6), indicating that phosphorylation sites were more likely to be exposed to the protein surface (*p* = 9.073E-04).

### Interaction Networks of Identified Phosphoproteins in *A. flavus*

**Figure 4:**
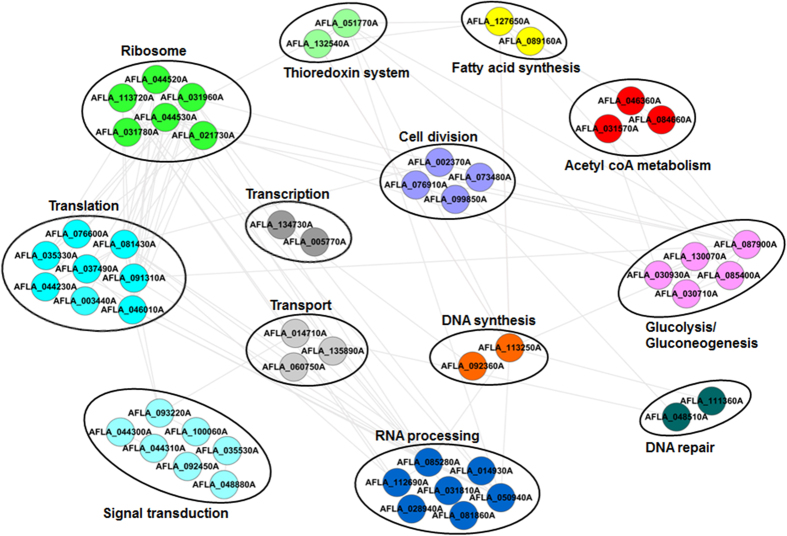
Protein–protein interaction network of a number of phosphorylated proteins identified in this study. The interaction network contains 53 nodes and 109 edges, as analyzed with String and adjusted with Cytoscape.

We next sought to establish protein interaction networks to identify the physical and functional interactions among the identified phosphoproteins, using protein interaction information from the String database. In total, 89 phosphoproteins and 236 edges were mapped to the protein interaction database (Supplementary Table S7). Then, functional category was used to group phosphoproteins, and interaction networks were then visualized with Cytoscape^29^, showed in Fig. 4. In the portion of global phosphoprotein interaction network, several complexes and cellular functions formed prominent, tightly connected clusters. As assessed by means of molecular complex detection, these included two proteins in DNA repair, two in DNA synthesis, two in transcription, two in fatty acid synthesis, two in the thioredoxin system, three in acetyl CoA metabolism, three in transport, four in cell division, five in glycolysis/gluconeogenesis, six in ribosome, seven in signal transduction, seven in RNA processing and eight in translation. It is widely accepted that acetyl-CoA is initial substrate of aflatoxins biosynthetic pathway, and is synthesized *via* oxidative decarboxylation of pyruvate and β-oxidation of fatty acids^1^. We found three phosphoproteins involved in acetyl CoA metabolism: acetyl-CoA carboxylase (ACC), pyruvate decarboxylase (PdcA), and 2-isopropylmalate synthase. Moreover, other two phosphoproteins (acyl carrier protein (ACP) and fatty acid synthase beta subunit (Fasβ)) were involved in fatty acid synthesis and five phosphoproteins (fructose-bisphosphate aldolase, phosphoglucomutase PgmA, phosphoglycerate mutase, hexokinase Kxk and pyruvate kinase) involved in glycolysis/gluconeogenesis, respectively. We anticipated that phosphorylation may affect the synthesis and metabolism of acetyl-CoA, consequently affecting aflatoxins formation directly or indirectly. These observations were most likely to reflect the importance of phosphorylation in aflatoxins formation. Moreover, it was important to note phosphorylation proteins involved in signal transduction pathways, including two SH3-domain-containing proteins, the 14-3-3 family protein ArtA, Ran-GTPase activating protein 1, Ran-specific GTPase-activating protein 1, MAPK kinase kinase Ste11 and serine/threonine kinase Ste20. Ste11 and Ste20 were involved in a MAPK signaling pathway, which could control the coordination of fungal development and secondary metabolite formation as previous report^30^. Therefore, it was easy to speculate that the interaction between these phosphoproteins might contribute to signal transduction and aflatoxins formation.

### Validation of Phosphoproteins by Western Blotting

**Figure 5:**
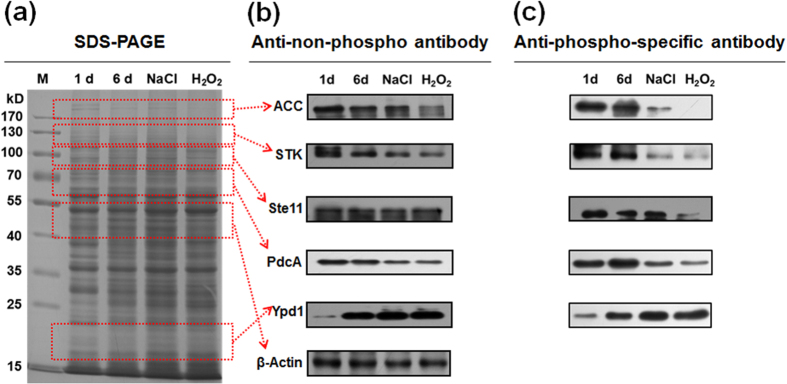
Immunoblotting verification of phosphorylated proteins. (**a**) An SDS-PAGE gel, stained with Coomassie Brilliant Blue. M, protein marker; 1d, protein obtained from *A. flavus* cultured for 1 d; 6 d, protein obtained from *A. flavus* cultured for 6 d; NaCl, protein obtained from *A. flavus* cultured for 6 d and exposed to 0.4 M NaCl for 24 h; H_2_O_2_, protein obtained from *A. flavus* cultured for 6 d and exposed to 0.4 mM H_2_O_2_ for 24 h. (**b**) Immunoblotting was performed using anti-non-phosphopeptide antibodies specific for acetyl-CoA carboxylase (ACC, protein molecular weight about 240 kD), serine/threonine protein kinase (STK, protein molecular weight about 125 kD), MAP kinase kinase kinase Ste11 (Ste11, protein molecular weight about 100 kD), pyruvate decarboxylase PdcA (PdcA, protein molecular weight about 65 kD), and phosphotransmitter protein Ypd1 (Ypd1, protein molecular weight about 20 kD). β-actin (about 43 kD) was used as control. To save on anti-non-phosphopeptide antibodies, PVDF membrane was cut based on molecular weight of different proteins. (**c**) Immunoblotting was performed using anti-phosphopeptide antibodies specific for ACC, STK, Ste11, PdcA and Ypd1. To save on anti-phosphopeptide antibodies, PVDF membrane was also cut based on molecular weight of different proteins. The gel in a and the blots in b and c were run under the same experimental conditions. The original pictures of full-length gels and blots were presented in the Supplementary Fig. S3.

To validate our phosphoproteomics findings and to determine whether environmental stimuli affect phosphorylation *in vivo*, the site-specific anti-phosphopeptide antibodies and anti-non-phosphopeptide antibodies were used to validate the phosphorylation status of five phosphoproteins, *viz.*, acetyl-CoA carboxylase ACC (S1138), pyruvate decarboxylase PdcA (S355), MAP kinase kinase kinase Ste11 (S187), phosphotransmitter protein Ypd1 (T14) and serine/threonine protein kinase (S153). As expected, the relative abundance of phosphoproteins differed depending on the different physiological/developmental stage of *A. flavus* (Fig. 5). The original pictures of full-length gel and blots were included in the Supplementary Fig. S3. This finding indicated that the phosphorylation status of proteins was affected by different physiological/developmental stages. We next sought to correlate changes in phosphoproteins at specific stages. Changes in the phosphorylation levels of acetyl-CoA carboxylase, pyruvate decarboxylase PdcA, MAP kinase kinase kinase Ste11 and phosphotransmitter protein Ypd1 were evident in the response of cells to a high-osmolarity stimulus (0.4 M NaCl), oxidative stress (0.4 mM H_2_O_2_) and during aflatoxins formation (6 d culture), as shown in Fig. 5. Notably, in contrast to the stage with no aflatoxins formation (1 d), we also observed the marked changes in phosphorylation of these proteins when cells were grown for 6 d. Furthermore, we detected the evident changes of the phosphorylation levels on these five identified proteins in response to oxidative stress and high-osmolarity stimulus, implying the potential connection between phosphorylation and aflatoxins biosynthesis. The reason is that previous studies have already proved that osmotic stress and oxidative stress play a regulated role in mycotoxins formation in filamentous fungi^31^,^32^. Based on the results, our data validated the presence of several phosphoproteins identified through our global phosphoproteomics approach and generated additional confidence in the accuracy of the MS data sets. In addition, the significant changes in protein phosphorylation status indicated that phosphorylation could play a key role in response to environmental stimuli and play an important role in aflatoxins formation.

### Phosphorylated Proteins Involved in Signaling Pathways and Aflatoxins Biosynthesis

**Figure 6:**
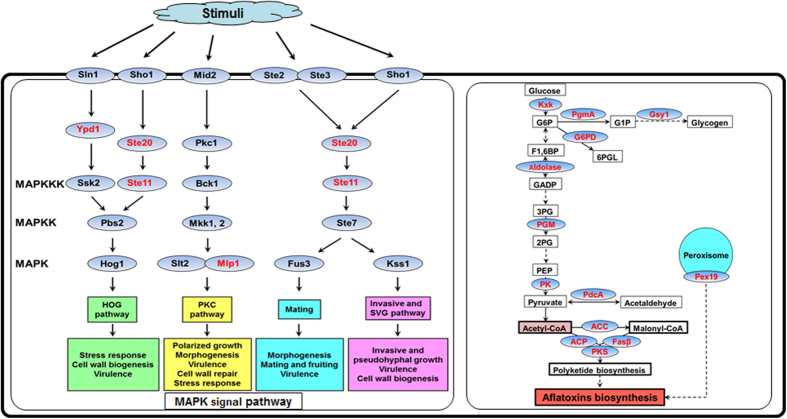
The schematic diagram indicating the participation of identified phosphoproteins (highlighted in red) in the MAPK signal pathway and in metabolism. Kxk: hexokinase Kxk. G6P: glucose-6-phosphate. PgmA: phosphoglucomutase PgmA. G1P: glucose-1-phosphate. Gsy1: glycogen synthase Gsy1. G6PD: glucose-6-phosphate 1-dehydrogenase. 6PGL: 6-phosphogluconolactone. F1,6BP: fructose 1,6-bisphosphate. Aldolase: fructose-bisphosphate aldolase. GADP: glyceraldehyde 3-phosphate. 3PG: 3-phosphoglycerate. PGM: phosphoglycerate mutase. 2PG: 2-phosphoglycerate. PEP: phosphoenolpyruvate. PK: pyruvate kinase. PdcA: pyruvate decarboxylase PdcA. ACC: acetyl-CoA carboxylase. ACP: acyl carrier protein. Fasβ: fatty acid synthase beta subunit. PKS: polyketide synthase. Pex19: peroxisomal membrane protein receptor Pex19. Ste11: MAP kinase kinase kinase Ste11. Ypd1: phosphorelay intermediate protein Ypd1. Ste20: serine/threonine kinase Ste20. Mlp: filament-forming protein Tpr/p270. Sho1: high osmolarity signaling protein Sho1. Sln1: sensor histidine kinase/response regulator TcsB/Sln1. Ste2: developmental regulator FlbA. Ste3: a-pheromone receptor PreA. Mid2: mating pheromone-induced death protein 2. Ste7: MAP kinase kinase Ste7. Fus3: MAP kinase Fus3. Kss1: MAP kinase Kss1. Ssk2: MAP kinase kinase kinase SskB. Pbs2: MAP kinase kinase Pbs2. Hog1: MAP kinase SakA. Pkc1: protein kinase c. Bck1: MAP kinase kinase kinase Bck1. Mkk1, 2: MAP kinase kinase Mkk2. Slt2: MAP kinase MpkA.

The large group of phosphoproteins observed in this study could yield new insights into metabolic pathways of the model pathogenic fungus *A. flavus*. Therefore, we assigned all identified phosphoproteins to KEGG pathways to evaluate whether the phosphoproteins played a role in any biological/signaling pathways. As shown in Supplementary Table S8, many identified phosphoproteins were involved in pathways responsible for the control of key physiological processes. For instance, 49 phosphorylated proteins were found to be involved in metabolic pathways and 16 were involved in biosynthesis of secondary metabolites. Importantly, we also identified a large proportion of phosphoproteins involved in MAPK signaling pathway and related to aflatoxins biosynthesis pathway. It is widely accepted that the MAPK signaling pathway is involved in environmental adaptation to various stresses, including osmotic stress, oxidative stress, heat shock, and pheromones^32^ and plays a pivotal role in the production of virulence in fungal pathogens by receiving and transmitting external signals from the plasma membrane to the nucleus^33^. In this study, we found that four phosphorylated proteins (MAP kinase kinase kinase Ste11, phosphorelay intermediate protein Ypd1, serine/threonine kinase Ste20 and filament-forming protein Tpr/p270) were mapped to the MAPK signaling pathway (Fig. 6). We anticipated that phosphorylation may regulate the activities of these proteins to affect the signal transduction in *A. flavus* as previously described^16^. Furthermore, increasing evidences show that the first steps in aflatoxins biosynthesis require formation of the starter unit hexanoate from the precursors acetyl-CoA synthesized *via* oxidative decarboxylation of pyruvate, β-oxidation of fatty acids or from ketogenic amino acids, in primary metabolism^1^,^34^,^35^. As shown in Fig. 6, the enzymes associated with oxidative decarboxylation of pyruvate (PdcA), β-oxidation of fatty acids (polyketide synthase, Fasβ and ACP), glycolysis/gluconeogenesis (glycogen synthase Gsy1, phosphoglucomutase PgmA, hexokinase Kxk, fructose-bisphosphate aldolase, phosphoglycerate mutase and pyruvate kinase) and pentose phosphate pathway (glucose-6-phosphate 1-dehydrogenase) were found to be phosphorylated, indicating that reversible phosphorylation may also regulate the synthesis of acetyl-CoA, related to aflatoxins biosynthesis. Furthermore, in this work, we also identified a phosphorylated peroxisome protein Pex19, a soluble cytosolic peroxin which may act as receptor/chaperone for newly synthesized peroxisomal membrane proteins. Previous report has demonstrated that the early steps in aflatoxins biosynthesis may occur in peroxisomes^36^ and Pex19 plays a key role in the reception of intracellular oxidative stress signals and transducing the oxylipin signals, which are needed for the progression of toxin synthesis^37^,^38^. Consequently, it was easy to associate that the reversible phosphorylation may affect the toxin biosynthesis by regulating the structure and function of Pex19.

### Effect of Phosphorylation of Ste11 on Growth, Conidia and Sclerotial Development

**Figure 7:**
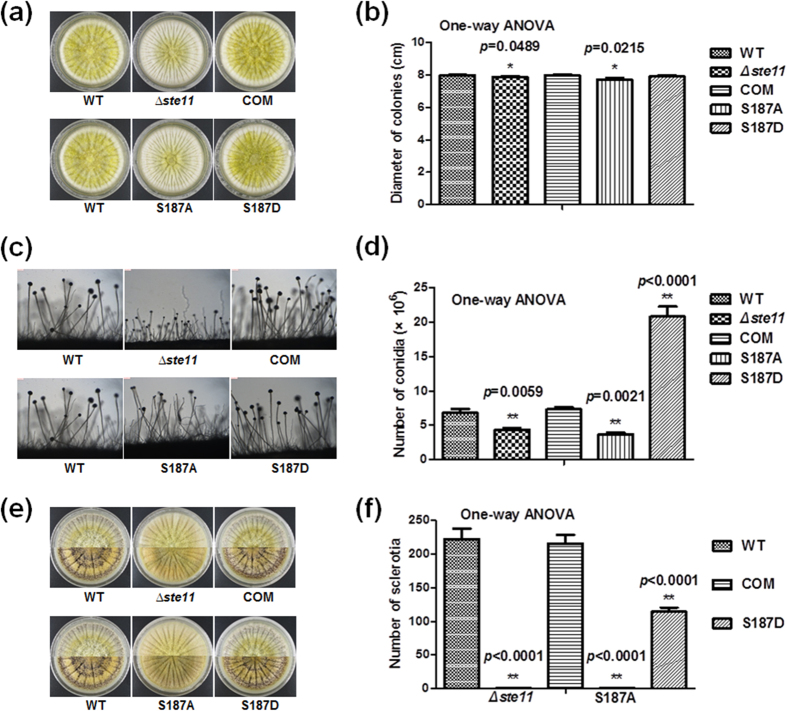
Phenotypic analyses of WT, *∆ste11*, COM, S187A and S187D mutants. (**a**) Colonial morphology. (**b**) Column diagram of colonial diameter. (**c**) Morphology of conidiophores. (**d**) Number of spore generation. (**e**) Morphology of sclerotial production. (**f**) Quantification of sclerotia. The asterisks *represents a significant difference level of *p*-value < 0.05 and asterisks **represents a significant difference level of *p*-value < 0.01. Error bars indicate SD from 3 independent experiments.

According to previously reported model that preactivated Ste20 initiates the kinase cascade system by phosphorylating the mitogen-activated protein kinase kinase kinase (MAPKKK) Ste11, it is conceivable that phosphorylation plays a vital role in the signal transduction^39^. In fungus *Aspergillus nidulans*, deletion of the MAPKK gene, *steC*, a homologue of *A. flavus ste11*, results in a slower growth rate, the formation of more branched hyphae, altered conidiophore morphology, an inhibition of heterokaryon formation and a block of cleistothecium development^40^. In our study, two reliable phosphorylation sites of Ste11 were identified. Therefore, we further assessed whether reversible phosphorylation at these positions would affect the function of Ste11. We constructed the *ste11* gene mutagenesis (*Δste11*), complemental strain (COM) and site-directed mutagenesis (S187A and S187D) of phosphorylation site of Ste11. The identified phosphorylation site 187^th^ S was converted to a non-phosphorylatable alanine (A) and to aspartic acid (D) to mimic a constitutively phosphorylation state. All mutations were verified by PCR or DNA sequencing (Supplementary Fig. S4) and then the effects of phosphorylation on Ste11 were measured. Consistent with previous report^40^, we found that different colonial morphology and smaller colonial diameter in *Δste11* strain and S187A mutant were detected, as compared with the WT strain, COM strain and S187D mutant when the strains were grown on YES media for 4 d (Fig. 7a,b). Accordingly, the *Δste11* strain and S187A mutant produced short conidiophores and fewer conidia on YES media (Fig. 7c,d). It was noteworthy that no sclerotium was produced in *Δste11* and S187A mutant when these strains were grown on Wickerham media for 10 d (Fig. 7e,f). Consequently, our results showed that Ste11 phosphorylation affected the colonial morphology, conidiophore, conidial formation and sclerotial production in *A. flavus*.

### Involvement of Phosphorylation in Aflatoxins Biosynthesis in *A. flavus*

**Figure 8:**
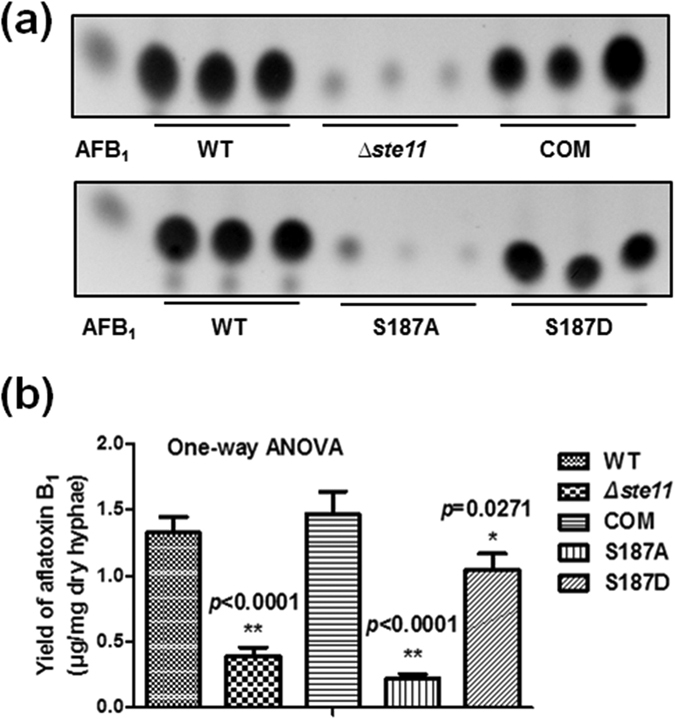
Aflatoxins formation of WT, *∆ste11*, COM, S187A and S187D mutants. (**a**) TLC analysis of aflatoxin B_1_ production. (**b**) Quantification of aflatoxin B_1_. The asterisks *represents a significant difference level of *p*-value < 0.05 and asterisks **represents a significant difference level of *p*-value < 0.01. Error bars indicate SD from 3 independent experiments.

Based on previous reports^18^,^36^ and our TLC analysis (Supplementary Fig. S1), the aflatoxin B_1_ level would increase when strain was grown into the stationary phase that led to nutritional deficiency. Recent study has also revealed the stress response linkage to the production of natural products, including mycotoxins^31^. In addition, it has been reported that the MAP kinase controls the stress response and regulates gliotoxin biosynthesis in *Aspergillus fumigatus*^41^. We speculated that the environmental stimuli may induce the aflatoxins biosynthesis in *A. flavus* by regulating the MAP kinase cascade system. To test this hypothesis, we performed the TLC analysis of the WT, *Δste11*, COM, S187A and S187D. Compared with the WT strain, the production of aflatoxin B_1_ in *Δste11* strain and S187A mutant were decreased memorably, whereas COM strain and S187D mutant had similar yield of aflatoxin B_1_ (Fig. 8). Our findings provided evidences that reversible phosphorylation affected the function of MAP kinase and the aflatoxins biosynthesis in *A. flavus*.

## Discussion

Here, to study the effect of phosphorylation on aflatoxins biosynthesis in *A. flavus*, we performed the first phosphoproteome of *A. flavus*. Totally, 598 high-confidence phosphorylation sites in 283 phosphoproteins were identified. According to GO annotation analysis, enrichment analysis, KEGG pathway prediction and the interaction network of this novel phosphoproteome, phosphorylated proteins in *A. flavus* were involved in a variety of important biological processes, including signal transduction and metabolic pathways, to perform numerous roles. Based on motif analysis, secondary structure and kinase prediction of phosphorylation sites, more informations about phosphorylation in *A. flavus* were obtained. It was helpful to further study the roles of phosphorylation in *A. flavus* and to prepare anti-phosphorylated antibody.

Based on bioinformatics analysis results of *A. flavus* phosphoproteome, it was obvious that phosphorylation was tightly connected with aflatoxins production. Firstly, acetyl CoA metabolism was controlled by phosphorylated proteins (Figs 4 and 6 and Supplementary Table S7). It is widely accepted that acetyl CoA is the initial substrate of aflatoxins biosynthesis and fatty acid synthesis^1^. In our study, phosphorylated proteins could regulate the flux of acetyl CoA and influence aflatoxins production. Additionly, calmodulin was identified with phosphorylation in phosphoproteome of *A. flavus* (Supplementary Table S1). The phosphorylation status of calmodulin regulated the activity of acetyl-CoA carboxylase and other regulatory enzymes to affect aflatoxins biosynthesis^15^,^42^. It is another powerful evidence of phosphorylation involved in aflatoxins production. Moreover, the phosphorylated protein Pex19 has been proved to be needed for the progression of toxin synthesis^37^,^38^ (Fig. 6). Needless to say, the phosphorylated proteins in signal transduction pathway, MAPK pathway, could transmit extracellular signals to regulate aflatoxins biosynthesis, proved by both previous studies^30^,^40^ (Fig. 6). The western blotting results of phosphorylated proteins further confirmed that phosphorylation was involved in aflatoxins production (Fig. 5).

To further reveal roles of phosphorylation in aflatoxins biosynthesis, we constructed deletion, complementation and point mutation strains of phosphorylated protein, Ste11. The results of phenotypic analyses demonstrated that phosphorylation played a crucial role in maintaining the growth, conidial production, sclerotial formation and aflatoxin biosynthesis (Figs 7 and 8). Ste11 is the MAPKKK in MAPK pathway. Based on morphological analyses, we speculated that phosphorylation may activate Ste11 to initiate the kinase cascade system, and subsequently the activation of Ste11-dependent stress signaling would influence colony, the initiation of conidia and sclerotial biosynthesis. Our results provide the first evidence linking the phosphorylation of MAP kinase with growth, conidial production and sclerotial formation in *A. flavus*.

To our knowledge, the MAPK cascade module was well conserved in all eukaryotes, playing a fundamental role in the regulation of various environmental responses *via* activating protein phosphorylation. The fungal MAPK module controls fungal development and secondary metabolite production^30^. The cascade was composed of three kinases: MAPKKK, mitogen-activated protein kinase kinase (MAPKK) and MAPK. MAPKK was activated *via* phosphorylation by MAPKKK, then activated MAPKK in turn activates and phosphorylates MAPK^43^. The activated MAPK were served to transduce signals to the nucleus, resulting in transcriptional responses^44^,^45^. In MAPK cascade of *A. nidulans*, the information is transmitted as phosphate signal from SteC to Fus3, which is in a complex with other proteins of the MAPK module. Then Fus3 phosphorylates and activates VeA protein, which is required for sexual development and coordinated secondary metabolite production. It is noteworthy that the *steC* deletion mutant showed a significant decrease of the sterigmatocystin (the precursor of aflatoxins biosynthesis) in *A. nidulans*^30^. In our study, the aflatoxins production of point mutation strain mimicked non-phosphorylation status (S187A) was consistent with that of *∆ste11*. Without phosphorylation, we speculated that Ste11 could not transmit phosphate signal. Hence, downstream Fus3 and VeA could be not activated. Finaly, aflatoxins biosynthesis was suppressed. It is convincible that phosphorylation may play an important role in regulating the aflatoxins biosynthesis in *A. flavus*.

Taken together, it is safe to say that phosphorylation is involved in aflatoxins biosynthesis.

## Conclusions

In conclusion, we presented a comprehensive phosphoproteome analysis of the model pathogenic fungus *A. flavus*. Phosphorylated proteins in *A. flavus* were involved in a variety of biological processes. Further functional studies demonstrated that phosphorylation may be a mechanism involved in aflatoxin biosynthesis in *A. flavus*. Therefore, our results provided a foundation for functional analyses of the phosphorylated proteins and opened an avenue toward better understanding of signaling pathways in this pathogen.

## Materials and Methods

### Strain, Media and Cultivation Conditions

*A. flavus* NRRL 3357, *A. fumigatus* Af293 and *A. flavus* CA14*Δku70ΔpyrGΔniaD* were obtained from Prof. Zhumei He (Sun Yat-Sen University, Guangzhou, China), Dr. Yang Liu (Institute of food science and technology, Chinese Academy of Agricultural Sciences, Beijing, China) and Dr. Ana M. Calvo (Northern Illinois University, Illinois, USA), respectively. Conidia of *A. flavus* NRRL 3357 were cultivated in 150 mL liquid YES media^46^ and shaked at 180 r/m in the dark for 7 d at 28 °C to extract proteins or aflatoxins^47^ (10^6^ conidia/mL liquid media)^48^. *A. fumigatus* Af293 was cultivated with YES agar statically in the dark at 28 °C to extract DNA and amplify *pyrG. A. flavus* CA14*Δku70ΔpyrGΔniaD* was cultivated with YGTUU agar^49^ statically in the dark at 28 °C for protoplasts preparation.

### Thin Layer Chromatography (TLC) Analysis

Cultures were added with equal volume of chloroform and agitated for 30 min. Aflatoxins dissolved in the chloroform were collected *via* centrifugation and separated using a separatory funnel. All samples were dried and then resuspended in chloroform. Then, 5 μL of each extract was loaded onto a silica gel TLC plate (200 × 200 mm, GF254, Qingdao Haiyang Chemical Co.), and the extracts were separated using acetone: chloroform (10: 90, v/v). Photographs were captured following exposure to UV radiation at 312 nm wavelength. The intensity of the samples was analyzed using a JD-801 Computer-aided Image Analysis System (JEDA Co.) by visual comparison with standard aflatoxin B_1_.

### Protein Extraction

Conidia of *A. flavus* NRRL 3357 were inoculated into liquid YES media and cultivated for 1 d or 6 d. Subsequently, a phosphatase inhibitor solution (NaF, Na_3_VO_4_ and Na_4_P_2_O_7_)^50^ and a protease inhibitor (PMSF, Beyotime) were added to the cultures at final concentrations of 10, 1, 10 and 1 mM, respectively; then, the cultures were agitated for 30 min. The hyphae were harvested by filtration and washed twice with PBS. After grinding the residue into powder, the pellets were resuspended in RIPA lysis buffer (Beyotime) containing PMSF and phosphatase inhibitors and incubated for 1 h at 4 °C. Cellular debris was removed by centrifugation at 7,000 × *g* for 20 min at 4 °C, and the supernatant was pipetted into Millipore Amicon Ultra-15 Centrifugal Filters (3 kD, Merck Millipore) to remove pigment and other small molecules with centrifugation at 4,000 × *g* for 30 min at 4 °C; this purification process was repeated twice until the lower liquid was colorless. Finally, the entire retentate fraction was transferred into a new centrifuge tube for further processing. Protein concentrations were determined with a BCA Protein Assay Kit (Tiangen).

### In-solution Digestion and Prefractionation

Protein extracts (20 mg) were subjected to disulfide reduction, alkylation and digestion as described previously^12^,^50^. Disulfide reduction was performed with 25 mM of DTT (37 °C, 45 min). Then alkylation was performed with 50 mM of iodoacetamide (25 °C, 20 min in the dark). Proteins were digested with sequencing grade trypsin (1:100 w/w) (Promega) at 37 °C for 4 h, and further digested with trypsin (1:100 w/w) at 37 °C for other 20 h. Then digestion was quenched with 0.1% trifluoroacetic acid (TFA). Then peptides were fractionated on self-packed AGT Cleanert SPE columns packed with C_18_ material (40 μm, 60-Å pore size; Agilent Technologies). The columns were washed three times with 15 mL of 100% acetonitrile (ACN) and then equilibrated three times with 15 mL of 20 mM ammonium formate. After the digested solution was loaded sequentially onto the self-packed C_18_ columns three times, the columns were washed with 5 mL of 20 mM ammonium formate, for sample desalting, and eluted with a series of elution buffers (2 mL) composed of 20 mM ammonium formate and different concentrations of ACN (10, 15, 18, 21, 25, 28, 35, 60, 80, and 100%). Fractions were collected in 2 mL centrifuge tubes, concentrated to less than 50 μL, and stored at −80 °C for further use.

### Enrichment of Phosphopeptides with TiO_2_ Resin

Phosphopeptides were enriched using a Phosphopeptide Enrichment TiO_2_ Kit (Calbiochem) with slight modifications of the manufacturer’s protocol. Specifically, the fractions were redissolved in 200 μL of TiO_2_ phosphobind buffer containing 50 g/L 2,5-dihydroxybenzoic acid. Then, 50 μL of TiO_2_ phosphobind resin was added to the mixtures, and then incubated on an oscillator. After 1 h incubation, mixtures were centrifuged, and the supernatant was discarded. Subsequently, the TiO_2_ phosphobind resin was washed three times with wash buffers I and II. Thereafter, phosphopeptides bound to the resin were eluted twice with 0.5% ammonium solution (pH 10.5) in 50% ACN. The eluates were combined and concentrated. Then, the combined eluates were concentrated to dryness and redissolved in 25 μL of an equal volume of 0.1% TFA and 5% ACN for future processing.

### LC-MS/MS Analysis

Phosphopeptides were separated on an Eksigent NanoLC-Ultra^®^ binary pump system with tray cooling and analyzed online using a Triple TOF™ 5600 Mass Spectrometer (AB SCIEX). The samples were delivered to a Nano cHiPLC Trap Column (200 μm × 0.5 mm ChromXP C_18_-CL; 3 μm; 120 Å) and then trapped on a Nano cHiPLC Column (75 μm × 15 cm ChromXP C_18_-CL; 3 μm; 120 Å). Peptides were eluted with a linear solvent gradient (described in Supplementary Methods). The ion spray voltage was set at 2300 V, declustering potential at 100 V, curtain gas flow at 30, and nebulizer gas 1 at 2. Information-dependent acquisition employed a 250 ms survey scan and up to 40 production scans, at a rate of 50 ms/per scan. Surveys of full scan MS spectra (from m/z 300 to 1,500) were acquired with a resolution of 40,000 for both MS and MS/MS. The 50 most intense ions were sequentially isolated for fragmentation in the quadrupole by collision-induced dissociation.

Enriched phosphopeptides were also separated on an Ultimate^TM^ 3000 nano-LC System (Thermo Scientific™ Dionex™) in tandem with an electro-spray ion-trap mass spectrometer HCT Ultra (Bruker Daltonics). The methods used for sample loaded, peptide elution from the column and the settings for the mass spectrometer were as described by Yang *et al.*^12^.

### Bioinformatics Analysis

The identified phosphoproteins were group into biological process, molecular function and cellular compartment based on gene ontology (GO) term by Blast2GO software^51^. The subcellular localization of the identified phosphorylated proteins was analyzed by YLoc (http://abi.inf.uni-tuebingen.de/Services/YLoc/webloc.cgi)^52^. DAVID (http://david.abcc. ncifcrf.gov/tools.jsp) was used to perform interpro and GO term enrichment analyses^53^. Motif specificities of phosphorylation sites were analyzed using an in-house script as previously reported^12^ and Motif-X (http://motif-x.med.harvard.edu/motif-x.html)^22^. Secondary structures of identified phosphoproteins were predicted with NetSurfP (http://www.cbs.dtu.dk/services/NetSurfP/)^26^ and the *p*-values of the identified phosphorylated residues were calculated as described by Wagner *et al.*^54^. Kinases targeting all identified phosphorylation sites were predicted with NetworKIN (http://networkin.info/version_2_0/newPrediction.php)^55^. Interaction network analysis was performed with String website (http://string-db.org/)^56^. The interaction network was visualized by Cytoscape (version 3.0.2)^29^.

### Western Blotting

Proteins from *A. flavus* NRRL 3357 were denatured in protein-loading buffer and separated by 12% SDS-PAGE. After electrophoresis, gels were stained with Coomassie Brilliant Blue or transferred to PVDF membranes (GE Healthcare). Immunoblotting was performed with the generated anti-phospho-site-specific antibodies (1:1000 dilution) or anti-non-phosphopeptide antibodies (1:1000 dilution), followed by incubation of the blots with a peroxidase-conjugated secondary antibody (1:5000 dilution; KPL). As a loading control, monoclonal mouse anti-actin antibodies were used. Membranes were washed three times with TBST, and proteins were detected by chemiluminescence using SuperSignal^®^ West Pico Chemiluminescent Substrate (Thermo Fisher Scientific). The results of western blotting were recorded with ImageQuant TL (GE Healthcare).

### Construction of *ste11* Mutants

The *A. flavus ste11* gene orthologs and *A. fumigatus* Af293 *pyrG* gene ortholog were identified, searched and downloaded from the *Aspergillus* Comparative Database (http://www.broadinstitute.org/annotation/genome/aspergillus_group/MultiHome.html). The full-length replacement mutation of *ste11* gene was introduced by a previous description^49^. The primers used in this study were showed in Supplementary Table S9. Chromosomal Integrating Shuttle Vector pPTR I (TAKARA) was used to build complementation vectors. *ste11* gene (including upstream promoter element and downstream 3′-non-translated region) was amplified and digested (Kpn I, Hind III, Thermo) and attached to digested pPTR I with ClonExpress II One Step Cloning Kit (C112-01, Vazyme Biotech Co.). Then complementation vector was transformed into protoplasts (gene deletion strain, ∆*ste11*) to construct complementation strain (COM), filtered by pyrithiamine resistance gene. The complementation vector was also used as the template of point mutant vectors (S187A and S187D). DpnI was used to eliminate template (complementation vector), and then point mutant vectors were transformed into protoplasts (∆*ste11*), filtered by pyrithiamine resistance gene. *A. fumigatus* Af293 *pyrG* gene was amplified and transformed into protoplasts (*A. flavus* CA14*Δku70ΔpyrGΔniaD*), used as wild type (WT).

### Phenotypic Analysis

10^4^ spores of WT, ∆*ste11*, COM, S187A and S187D were point inoculated onto YES agar, cultivated for 4 d. Colonial phenotypes were recorded. Conidia were collected with 7% DMSO and 0.5% Tween-20 and counted using a hemocytometer. Conidial formation was observed as described previously^49^. For sclerotial analysis, 10^4^ conidia of each strain were spread on Wickerham media plates and statically incubated at 37 °C for 10 d in the dark. Plates were sprayed with 70% ethanol to kill and wash away conidia to aid in enumeration of sclerotia. Each experiment was performed with four replicates for three times.

## Additional Information

**How to cite this article**: Ren, S. *et al.* Global Phosphoproteomic Analysis Reveals the Involvement of Phosphorylation in Aflatoxins Biosynthesis in the Pathogenic Fungus *Aspergillus flavus. Sci. Rep.* **6**, 34078; doi: 10.1038/srep34078 (2016).

## Supplementary Material

Supplementary Information

Supplementary Table S1

Supplementary Table S2

Supplementary Table S3

Supplementary Table S4

Supplementary Table S5

Supplementary Table S6

Supplementary Table S7

Supplementary Table S8

## References

[b1] Yabe K. & Nakajima H. Enzyme reactions and genes in aflatoxin biosynthesis. Applied Microbiology and Biotechnology 64, 745–755, doi: 10.1007/s00253-004-1566-x (2004). 15022028

[b2] Khlangwiset P., Shephard G. S. & Wu F. Aflatoxins and growth impairment: a review. Critical Reviews in Toxicology 41, 740–755, doi: 10.3109/10408444.2011.575766 (2011). 21711088

[b3] Hunter T. Signaling-2000 and beyond. Cell 100, 113–127, doi: 10.1016/S0092-8674(00)81688-8 (2000). 10647936

[b4] van Bentem S. D., Mentzen W. I., de la Fuente A. & Hirt H. Towards functional phosphoproteomics by mapping differential phosphorylation events in signaling networks. Proteomics 8, 4453–4465, doi: 10.1002/pmic.200800175 (2008). 18972525

[b5] Preisinger C., von Kriegsheim A., Matallanas D. & Kolch W. Proteomics and phosphoproteomics for the mapping of cellular signalling networks. Proteomics 8, 4402–4415, doi: 10.1002/pmic.200800136 (2008). 18846508

[b6] Bogoyevitch M. A. & Court N. W. Counting on mitogen-activated protein kinases-ERKs 3, 4, 5, 6, 7 and 8. Cellular Signalling 16, 1345–1354, doi: 10.1016/j.cellsig.2004.05.004 (2004). 15381250

[b7] Cohen P. Protein kinases-the major drug targets of the twenty-first century? Nature Reviews Drug Discovery 1, 309–315, doi: 10.1038/nrd773 (2002). 12120282

[b8] Pinkse M. W. H. *et al.* Highly robust, automated, and sensitive on line TiO_2_-based phosphoproteomics applied to study endogenous phosphorylation in *Drosophila melanogaster*. Journal of Proteome Research 7, 687–697, doi: 10.1021/pr700605z (2008). 18034456

[b9] Villen J., Beausoleil S. A., Gerber S. A. & Gygi S. P. Large-scale phosphorylation analysis of mouse liver. P Natl Acad Sci USA 104, 1488–1493, doi: 10.1073/pnas.0609836104 (2007). PMC1785252 17242355

[b10] Olsen J. V. *et al.* Global, *in vivo*, and site-specific phosphorylation dynamics in signaling networks. Cell 127, 635–648, doi: 10.1016/j.cell.2006.09.026 (2006). 17081983

[b11] Bodenmiller B. *et al.* Phosphoproteomic analysis reveals interconnected system-wide responses to perturbations of kinases and phosphatases in yeast. Science Signaling 3, doi: 10.1126/scisignal.2001182 (2010). PMC3072779 21177495

[b12] Yang M. K. *et al.* Global phosphoproteomic analysis reveals diverse functions of serine/threonine/tyrosine phosphorylation in the model cyanobacterium *Synechococcus* sp. strain PCC 7002. Journal of Proteome Research 12, 1909–1923, doi: 10.1021/pr4000043 (2013). 23461524

[b13] Ramsubramaniam N., Tao F., Li S. W. & Marten M. R. Cost-effective isobaric tagging for quantitative phosphoproteomics using DiART reagents. Molecular Biosystems 9, 2981–2987, doi: 10.1039/c3mb70358d (2013). 24129742

[b14] Beltrao P. *et al.* Evolution of phosphoregulation: comparison of phosphorylation patterns across yeast species. Plos Biol 7, 11274–11279, doi: 10.1371/journal.pbio.1000134 (2009). PMC2691599 19547744

[b15] Jayashree T., Rao J. P. & Subramanyam C. Regulation of aflatoxin production by Ca^2+^/calmodulin-dependent protein phosphorylation and dephosphorylation. Fems Microbiology Letters 183, 215–219 (2000). 10675586 10.1111/j.1574-6968.2000.tb08960.x

[b16] Shimizu K., Hicks J. K., Huang T. P. & Keller N. P. Pka, Ras and RGS protein interactions regulate activity of *aflR*, a Zn(II)2Cys6 transcription factor in *Aspergillus nidulans*. Genetics 165, 1095–1104 (2003). 14668367 10.1093/genetics/165.3.1095 PMC1462812

[b17] Bhatnagar D., Ehrlich K. C. & Cleveland T. E. Molecular genetic analysis and regulation of aflatoxin biosynthesis. Applied Microbiology and Biotechnology 61, 83–93, doi: 10.1007/s00253-002-1199-x (2003). 12655449

[b18] Roze L. V., Arthur A. E., Hong S. Y., Chanda A. & Linz J. E. The initiation and pattern of spread of histone H4 acetylation parallel the order of transcriptional activation of genes in the aflatoxin cluster. Mol Microbiol 66, 713–726, doi: 10.1111/j.1365-2958.2007.05952.x (2007). 17919289

[b19] Komeili A. & O’Shea E. K. Roles of phosphorylation sites in regulating activity of the transcription factor Pho4. Science (New York, NY) 284, 977–980, doi: 10.1126/science.284.5416.977 (1999). 10320381

[b20] Jans D. A. & Hubner S. Regulation of protein transport to the nucleus: central role of phosphorylation. Physiological reviews 76, 651–685 (1996). 8757785 10.1152/physrev.1996.76.3.651

[b21] Onorato J. J. *et al.* Role of acidic amino-acids in peptide-substrates of the beta-adrenergic-receptor kinase and rhodopsin kinase. Biochemistry 30, 5118–5125, doi: 10.1021/bi00235a002 (1991). 1645191

[b22] Schwartz D. & Gygi S. P. An iterative statistical approach to the identification of protein phosphorylation motifs from large-scale data sets. Nat Biotechnol 23, 1391–1398, doi: 10.1038/nbt1146 (2005). 16273072

[b23] Pearson R. B. & Kemp B. E. Protein kinase phosphorylation site sequences and consensus specificity motifs: tabulations. Methods Enzymol 200, 62–81 (1991). 1956339 10.1016/0076-6879(91)00127-i

[b24] Cohen P. The regulation of protein function by multisite phosphorylation–a 25 year update. Trends in Biochemical Sciences 25, 596–601 (2000). 11116185 10.1016/s0968-0004(00)01712-6

[b25] Radivojac P., Chawla N. V., Dunker A. K. & Obradovic Z. Classification and knowledge discovery in protein databases. Journal of Biomedical Informatics 37, 224–239 (2004). 15465476 10.1016/j.jbi.2004.07.008

[b26] Petersen B., Petersen T. N., Andersen P., Nielsen M. & Lundegaard C. A generic method for assignment of reliability scores applied to solvent accessibility predictions. BMC Struct Biol 9, 51, doi: 10.1186/1472-6807-9-51 (2009). 19646261 PMC2725087

[b27] Iakoucheva L. M. *et al.* The importance of intrinsic disorder for protein phosphorylation. Nucleic Acids Res 32, 1037–1049, doi: 10.1093/nar/gkh253 (2004). 14960716 PMC373391

[b28] He D. *et al.* Global proteome analyses of lysine acetylation and succinylation reveal the widespread involvement of both modification in metabolism in the embryo of germinating rice seed. Journal of Proteome Research (2016). 10.1021/acs.jproteome.5b00805 26767346

[b29] Cline M. S. *et al.* Integration of biological networks and gene expression data using Cytoscape. Nat Protoc 2, 2366–2382, doi: 10.1038/nprot.2007.324 (2007). 17947979 PMC3685583

[b30] Bayram O. *et al.* The *Aspergillus nidulans* MAPK module AnSte11-Ste50-Ste7-Fus3 controls development and secondary metabolism. Plos Genetics 8, doi: 10.1371/journal.pgen.1002816 (2012). PMC3400554 22829779

[b31] Duran R., Cary J. W. & Calvo A. M. Role of the osmotic stress regulatory pathway in morphogenesis and secondary metabolism in filamentous fungi. Toxins 2, 367–381, doi: 10.3390/toxins2040367 (2010). 22069590 PMC3153207

[b32] Hong S. Y., Roze L. V. & Linz J. E. Oxidative stress-related transcription factors in the regulation of secondary metabolism. Toxins 5, 683–702, doi: 10.3390/toxins5040683 (2013). 23598564 PMC3705287

[b33] Roman E., Arana D. M., Nombela C., Alonso-Monge R. & Pla J. MAP kinase pathways as regulators of fungal virulence. Trends in Microbiology 15, 181–190, doi: 10.1016/j.tim.2007.02.001 (2007). 17321137

[b34] Mahanti N., Bhatnagar D., Cary J. W., Joubran J. & Linz J. E. Structure and function of *fas-1A*, a gene encoding a putative fatty acid synthetase directly involved in aflatoxin biosynthesis in *Aspergillus parasiticus*. Applied and Environmental Microbiology 62, 191–195 (1996). 8572694 10.1128/aem.62.1.191-195.1996 PMC167785

[b35] Minto R. E. & Townsend C. A. Enzymology and molecular biology of aflatoxin biosynthesis. Chemical reviews 97, 2537–2555, doi: 10.1021/Cr960032y (1997). 11851470

[b36] Chanda A. *et al.* A key role for vesicles in fungal secondary metabolism. P Natl Acad Sci USA 106, 19533–19538, doi: 10.1073/pnas.0907416106 (2009). PMC2773199 19889978

[b37] Narasaiah K. V., Sashidhar R. B. & Subramanyam C. Biochemical analysis of oxidative stress in the production of aflatoxin and its precursor intermediates. Mycopathologia 162, 179–189, doi: 10.1007/s11046-006-0052-7 (2006). 16944285

[b38] Tsitsigiannis D. I. & Keller N. P. Oxylipins as developmental and host-fungal communication signals. Trends in Microbiology 15, 109–118, doi: 10.1016/j.tim.2007.01.005 (2007). 17276068

[b39] Saito H. Regulation of cross-talk in yeast MAPK signaling pathways. Curr Opin Microbiol 13, 677–683, doi: 10.1016/j.mib.2010.09.001 (2010). 20880736

[b40] Wei H. J., Requena N. & Fischer R. The MAPKK kinase SteC regulates conidiophore morphology and is essential for heterokaryon formation and sexual development in the homothallic fungus *Aspergillus nidulans*. Mol Microbiol 47, 1577–1588, doi: 10.1046/j.1365-2958.2003.03405.x (2003). 12622813

[b41] Jain R. *et al.* The MAP kinase MpkA controls cell wall integrity, oxidative stress response, gliotoxin production and iron adaptation in *Aspergillus fumigatus*. Mol Microbiol 82, 39–53, doi: 10.1111/j.1365-2958.2011.07778.x (2011). 21883519 PMC3229709

[b42] Rao J. P. & Subramanyam C. Calmodulin mediated activation of acetyl-CoA carboxylase during aflatoxin production by *Aspergillus parasiticus*. Letters in Applied Microbiology 30, 277–281 (2000). 10792646 10.1046/j.1472-765x.2000.00717.x

[b43] Wilkinson M. G. & Millar J. B. Control of the eukaryotic cell cycle by MAP kinase signaling pathways. FASEB journal: official publication of the Federation of American Societies for Experimental Biology 14, 2147–2157, doi: 10.1096/fj.00-0102rev (2000). 11053235

[b44] Gustin M. C., Albertyn J., Alexander M. & Davenport K. MAP kinase pathways in the yeast *Saccharomyces cerevisiae*. Microbiology and Molecular Biology Review 62, 1264–1300 (1998). 10.1128/mmbr.62.4.1264-1300.1998 PMC98946 9841672

[b45] Treisman R. Regulation of transcription by MAP kinase cascades. Current opinion in cell biology 8, 205–215 (1996). 8791420 10.1016/s0955-0674(96)80067-6

[b46] Abdel-Hadi A., Schmidt-Heydt M., Parra R., Geisen R. & Magan N. A systems approach to model the relationship between aflatoxin gene cluster expression, environmental factors, growth and toxin production by *Aspergillus flavus*. Journal of the Royal Society Interface 9, 757–767, doi: 10.1098/rsif.2011.0482 (2012). PMC3284141 21880616

[b47] Calvo A. M., Bok J., Brooks W. & Keller N. P. *veA* is required for toxin and sclerotial production in *Aspergillus parasiticus*. Applied and Environmental Microbiology 70, 4733–4739, doi: 10.1128/Aem.70.8.4733-4739.2004 (2004). 15294809 PMC492383

[b48] Georgianna D. R., Hawkridge A. M., Muddiman D. C. & Payne G. A. Temperature-dependent regulation of proteins in *Aspergillus flavus*: whole organism stable isotope labeling by amino acids. Journal of Proteome Research 7, 2973–2979, doi: 10.1021/pr8001047 (2008). 18529071

[b49] Yang K. L. *et al.* The DmtA methyltransferase contributes to *Aspergillus flavus* conidiation, sclerotial production, aflatoxin biosynthesis and virulence. Scientific Reports 6, 23259, doi: 10.1038/srep23259 (2016). 26979781 PMC4793245

[b50] Chen Z. *et al.* Effects of phosphorylation of beta subunits of phycocyanins on state transition in the model cyanobacterium *Synechocystis* sp. PCC 6803. Plant and cell physiology 56, 1997–2013, doi: 10.1093/pcp/pcv118 (2015). 26315596

[b51] Conesa A. *et al.* Blast2GO: a universal tool for annotation, visualization and analysis in functional genomics research. Bioinformatics 21, 3674–3676, doi: 10.1093/bioinformatics/bti610 (2005). 16081474

[b52] Briesemeister S., Rahnenfuhrer J. & Kohlbacher O. Going from where to why-interpretable prediction of protein subcellular localization. Bioinformatics 26, 1232–1238, doi: 10.1093/bioinformatics/btq115 (2010). 20299325 PMC2859129

[b53] Huang da W., Sherman B. T. & Lempicki R. A. Bioinformatics enrichment tools: paths toward the comprehensive functional analysis of large gene lists. Nucleic Acids Res 37, 1–13, doi: 10.1093/nar/gkn923 (2009). 19033363 PMC2615629

[b54] Wagner S. A. *et al.* A proteome-wide, quantitative survey of *in vivo* ubiquitylation sites reveals widespread regulatory roles. Mol Cell Proteomics 10, doi: 10.1074/mcp.M111.013284 (2011). PMC3205876 21890473

[b55] Linding R. *et al.* Systematic discovery of *in vivo* phosphorylation networks. Cell 129, 1415–1426, doi: 10.1016/j.cell.2007.05.052 (2007). 17570479 PMC2692296

[b56] Franceschini A. *et al.* STRING v9.1: protein-protein interaction networks, with increased coverage and integration. Nucleic Acids Res 41, 808–815, doi: 10.1093/nar/gks1094 (2013). PMC3531103 23203871

