# Supplementary material for: Global Phosphoproteomic Analysis Reveals the Involvement of Phosphorylation in Aflatoxins Biosynthesis in the Pathogenic Fungus Aspergillus flavus
Source: Sci Rep. 2016 Sep 26;6:34078. doi: 10.1038/srep34078 (PMC5036175; doi:10.1038/srep34078)
Supplement: Supplementary Information [file srep34078-s1.pdf]

Global Phosphoproteomic Analysis Reveals the Involvement of Phosphorylation in Aflatoxins Biosynthesis in the Pathogenic Fungus *Aspergillus flavus*

Silin Ren[1#], Mingkun Yang[2#], Yu Li[1], Feng Zhang[1], Zhuo Chen[2], Jia Zhang[2], Guang Yang[1], Yuewei Yue[1], Siting Li[2], Feng Ge[2]*, Shihua Wang [1]*

[1] Key Laboratory of Pathogenic Fungi and Mycotoxins of Fujian Province and School of Life Sciences, Fujian Agriculture and Forestry University, Fuzhou, 350002, China

[2] Key Laboratory of Algal Biology, Institute of Hydrobiology, Chinese Academy of Sciences, Wuhan 430072, China

[#] These authors contributed equally to this work.

*To whom correspondence should be addressed: Prof. Shihua Wang, E-mail: wshyyl@sina.com, Tel & Fax: +86 591 87984471; Prof. Feng Ge, E-mail: gefeng@ihb.ac.cn, Phone/Fax: +86 27 68780500

Supplementary Methods

HPLC Gradient

Buffer A consisted of 98% water + 2% acetonitrile + 0.1% formic acid and buffer B consisted of 98% acetonitrile + 2% water + 0.1% formic acid. Peptides were eluted at a flow rate of 300 nL/min for 90 min using the following gradient: 1 min: 92% buffer A; 54 min: buffer B, with a gradient from 8% to 23%; 20 min: buffer B, with a gradient from 23% to 52%, 1 min: buffer B with a gradient from 52% to 80%; this was continued for 4 min to wash the column; then, a decreasing gradient of buffer B from 80% to 5% over 0.1 min; followed by 95% buffer A to equilibrate the column for the last 9.9 min.

Raw Data Analysis

The raw files from the AB SCIEX Triple TOF™ 5600 Mass Spectrometer were processed with ProteinPilot™ Software v.4.5 Beta and Scaffold, coupled with Mascot. The raw data files (.wiff) were converted into peak lists (mascot generic format; .mgf) and searched, using ProteinPilot™ Software, against *A. flavus* NRRL 3357 protein database (downloaded from http://www.ncbi.nlm.nih.gov/protein?LinkName=genome_protein&from_uid=360 in January 2013 and contained 13,485 protein sequences) concatenated with a reverse decoy database and protein sequences of common contaminants. Enzyme specifity was set as full cleavage by trypsin. Carbamidomethylation (Cys) was set as a fixed modification, whereas alkylation and phosphorylation were emphasized as special factors; biological modifications were entered as ID focus; and false discovery rate (FDR) analysis was set as <1%. Then, the .mgf files were searched against the *A. flavus* NRRL 3357 protein database with Mascot 2.3 (Matrix Science; Columbia, SC, USA) with the same parameters. All Mascot search results were loaded into

Scaffold (version 4.0.5, Proteome Software Inc., Portland, OR, USA) to validate MS/MS-based peptide and protein identifications.

The raw files from the Bruker Ion-Trap Mass Spectrometer were converted to .mgf files with DataAnalysis 4.0 (Bruker Compass; Fremont, CA, USA). Mascot 2.3 and pFind Studio 2.6 were used for further analyses[1-3]. The search criterion was trypsin digestion, with up to two missed cleavages. Fixed modification was carbamidomethylation (Cys), and variable modifications were oxidation (Met) and phosphorylation (S, T, Y). Precursor ion mass tolerance was set as ± 0.4 Da, and fragment ion mass tolerance was set as ± 0.6 Da. The threshold for the Mascot score was filtered at 1% FDR. To calculate the FDR, the target-decoy strategy was used, as determined by the equation $FDR = 2[n_{Decoy}/(n_{Decoy} + n_{Target})]$[4-6], and the threshold was determined at 1% FDR. If the E score of pFind was $<10^{-5}$ or if the Mascot score was >20, peptides were accepted. All raw data and the converted .mgf files have been uploaded to the publicly accessible database PeptideAtlas (dataset ID PASS00400) (http://www.peptideatlas.org/PASS/PASS00400)[7].

Localization of Phosphorylation Sites

All MS/MS spectra of phosphopeptides were manually verified using a previously described method[8,9]. The probabilities of phosphorylation at each potential site were evaluated using the Ascore algorithm in ScaffoldPTM for the results of ProteinPilot[10]. Phosphopeptides with an Ascore ⩾19 were accepted as a class one site. An Ascore ⩾19 was considered to indicate that the phosphorylation assignment had a 99% chance of being correct. For the remaining phosphopeptides without an Ascore, the phosphopeptides with a minimum of 95% confidence as determined by ProteinPilot were accepted, and the spectra were manually evaluated. In

addition, the accuracy of the localization of phosphorylation sites determined by Mascot and pFind was confirmed using probability-based PTM scores[11-13]. Phosphorylation sites with a probability of >0.75 were considered class one phosphorylation sites.

Production of Phosphorylation Site-specific Antibodies

The generations of polyclonal antibodies against *A. flavus* proteins were carried out by ABclonal Inc. (Wuhan, Hubei, China). Briefly, the polyclonal antibody of acetyl-coenzyme A carboxylase (ACC), pyruvate decarboxylase (PdcA), MAP kinase kinase kinase (Ste11), phosphotransmitter protein (Ypd1) and serine/threonine protein kinase (STK) were generated against the synthetic peptides STHPpSTPSTPTTESNPFR, MGNLNVGPVpSPPSN, RFSHTADNAHGYKPPSRPGpSPLR, TQTVQpTPPKTEGPATLDDMKEHIDK and RpSLSLPEVR, respectively. These unmodified synthetic peptides were used as control. Following purification of these antigens, immunization and sampling of the antisera from rabbit were performed by AB clonal, according to standard operating procedures. The specificity of the generated antibodies was determined by the manufacturer using ELISA and western blotting.

RNA Extraction

Mycelia of WT and all genetically engineered mutants were harvested and grinded with liquid nitrogen, and TRIzol reagent (Biomarker Technologies, Beijing, China) was used to isolate RNA. RNA was purified with the DNase (Thermo) and the first strand cDNA was synthesized with TransScript® All-in-One First-Strand cDNA Synthesis SuperMix (TransGen Biotech, Beijing, China).

References

1 Wang, L. H. *et al.* PFind 2.0: a software package for peptide and protein identification via tandem mass spectrometry. *Rapid Commun Mass Sp* **21**, 2985-2991, doi:10.1002/rcm.3173 (2007).

2 Fu, Y. *et al.* Exploiting the kernel trick to correlate fragment ions for peptide identification via tandem mass spectrometry. *Bioinformatics* **20**, 1948-1954, doi:10.1093/bioinformatics/bth186 (2004).

3 Li, D. Q. *et al.* pFind: a novel database-searching software system for automated peptide and protein identification via tandem mass spectrometry. *Bioinformatics* **21**, 3049-3050, doi:10.1093/bioinformatics/bti439 (2005).

4 Nesvizhskii, A. I., Vitek, O. & Aebersold, R. Analysis and validation of proteomic data generated by tandem mass spectrometry. *Nat Methods* **4**, 787-797, doi:10.1038/Nmeth1088 (2007).

5 Elias, J. E., Haas, W., Faherty, B. K. & Gygi, S. P. Comparative evaluation of mass spectrometry platforms used in large-scale proteomics investigations. *Nat Methods* **2**, 667-675, doi:10.1038/NMETH785 (2005).

6 Elias, J. E. & Gygi, S. P. Target-decoy search strategy for increased confidence in large-scale protein identifications by mass spectrometry. *Nat Methods* **4**, 207-214, doi:10.1038/nmeth1019 (2007).

7 Desiere, F. *et al.* The PeptideAtlas project. *Nucleic Acids Res* **34**, D655-D658, doi:10.1093/nar/gkj040 (2006).

8 Macek, B. *et al.* Phosphoproteome analysis of *E. coli* reveals evolutionary conservation of bacterial Ser/Thr/Tyr phosphorylation. *Mol Cell Proteomics* **7**, 299-307, doi:10.1074/mcp.M700311-MCP200 (2008).

9 Macek, B. *et al.* The serine/threonine/tyrosine phosphoproteome of the model bacterium *Bacillus subtilis*. *Mol Cell Proteomics* **6**, 697-707, doi:10.1074/mcp.M600464-MCP200 (2007).

10 Wu, X. R. *et al.* Investigation of receptor interacting protein (RIP3)-dependent protein phosphorylation by quantitative phosphoproteomics. *Mol Cell Proteomics* **11**, 1640-1651, doi:10.1074/mcp.M112.019091 (2012).

11 Peng, Y. J. *et al.* A RING-type E3 ligase controls anther dehiscence by activating the jasmonate biosynthetic pathway gene defective in anther dehiscence1 in *Arabidopsis*. *Plant J* **74**, 310-327, doi:10.1111/tpj.12122 (2013).

12 Olsen, J. V. & Mann, M. Improved peptide identification in proteomics by two consecutive stages of mass spectrometric fragmentation. *P Natl Acad Sci USA* **101**, 13417-13422, doi:10.1073/pnas.0405549101 (2004).

13 Olsen, J. V. *et al.* Global, in vivo, and site-specific phosphorylation dynamics in signaling networks. *Cell* **127**, 635-648, doi:10.1016/j.cell.2006.09.026 (2006).

Supplementary Figures and Supplementary Table

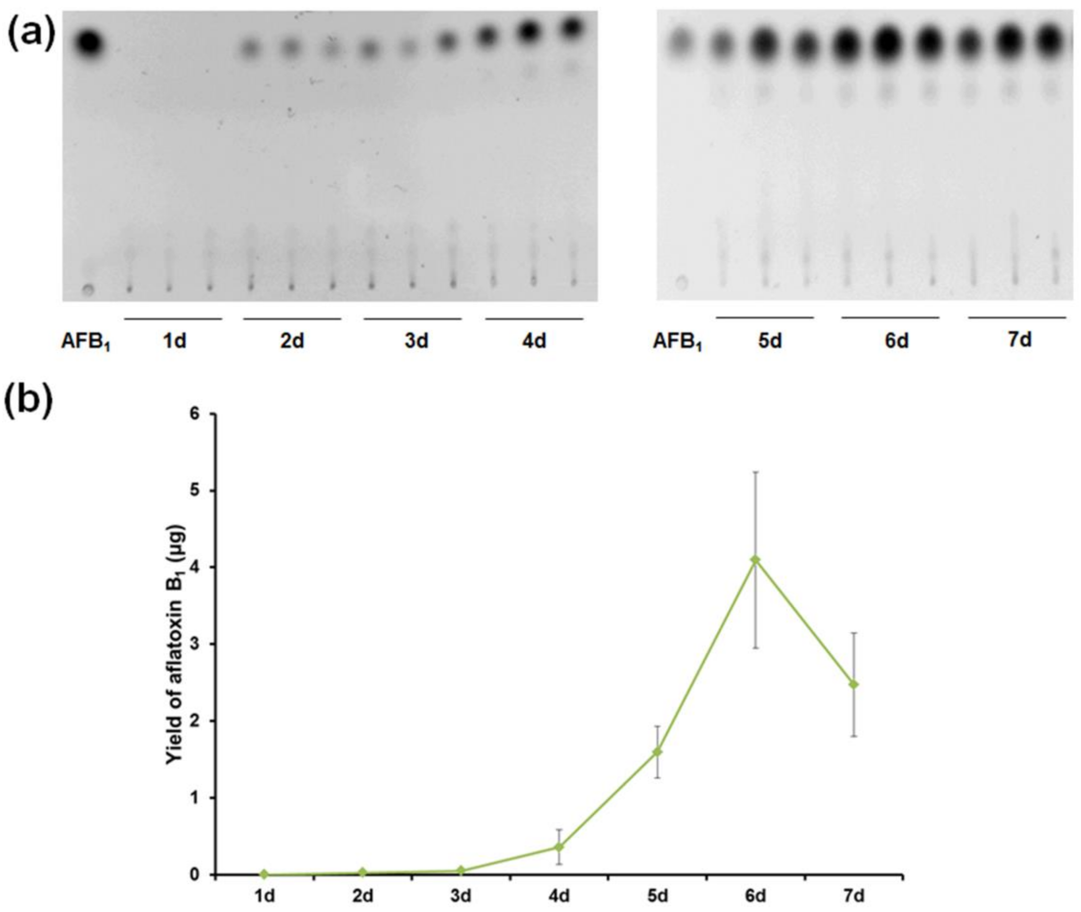

Supplementary Figure S1. Graphical representation of thin layer chromatography (TLC) results. (a) TLC separation of aflatoxin B_1 obtained from *A. flavus* after different culture periods. As control, 0.5 µg aflatoxin B_1 was loaded. (b) A line chart representing the results of continuous monitoring of aflatoxin B_1 production for 7 d, as quantified using a JD801 ImageQuant Scanner.

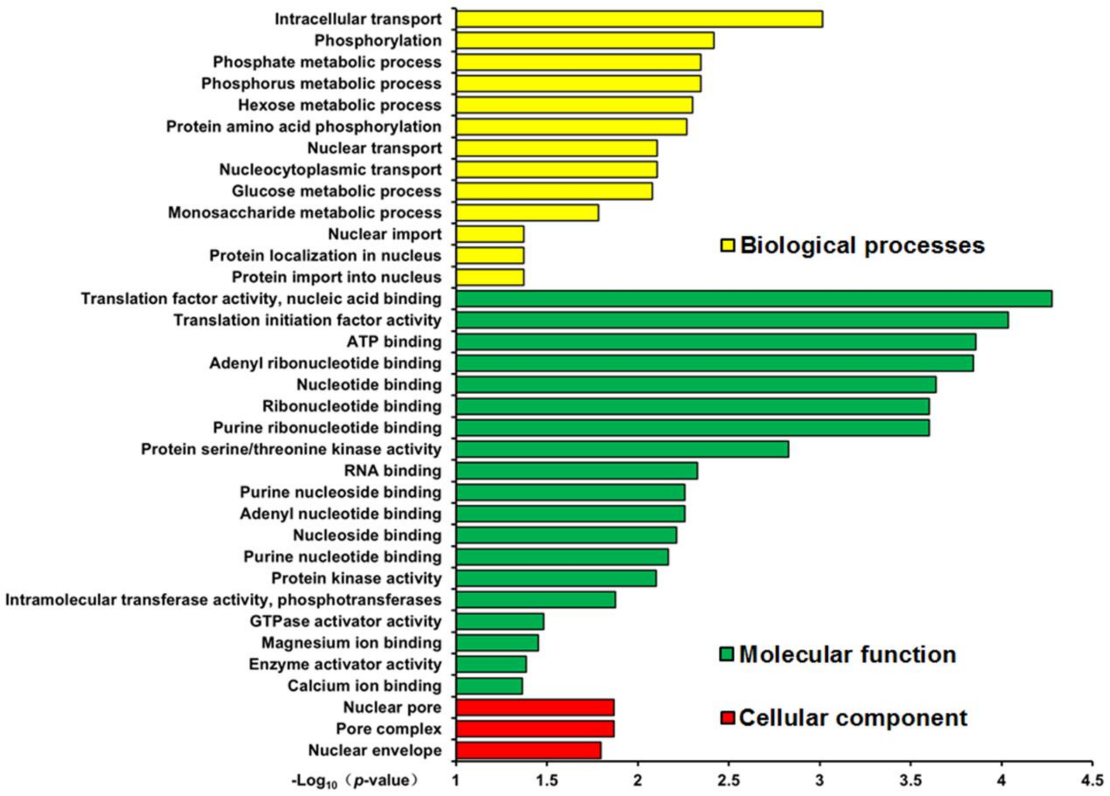

Supplementary Figure S2. Gene-annotation enrichment of phosphorylated proteins. The *x*-axis represents the negative value of Log_{10} of the *p*-value. Proteins with a *p*-value < 0.05 were considered significant.

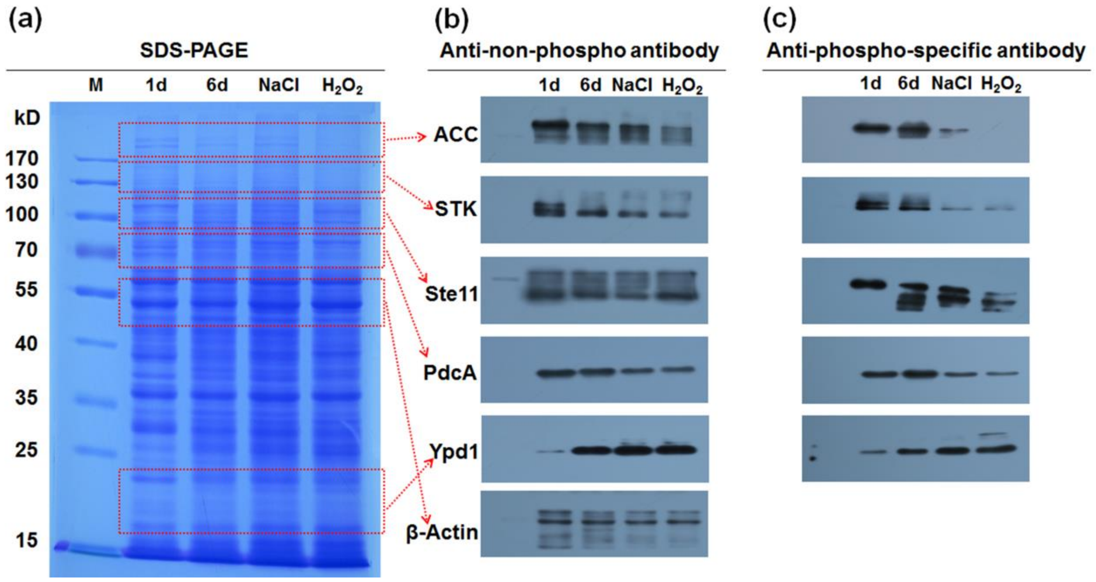

Supplementary Figure S3: The original picture of full-length gel and blots showed in **Figure 4**. (a) The SDS-PAGE gel, stained with Coomassie Brilliant Blue. M, protein marker; 1d, protein obtained from *A. flavus* cultured for 1 d; 6d, protein obtained from *A. flavus* cultured for 6 d; NaCl, protein obtained from *A. flavus* cultured for 6 d and exposed to 0.4 M NaCl for 24 h; H_2O_2, protein obtained from *A. flavus* cultured for 6 d and exposed to 0.4 mM H_2O_2 for 24 h. (b) The photographic films were performed using anti-non-phosphopeptide antibodies specific for acetyl-CoA carboxylase (ACC, protein molecular weight about 240 kD), serine/threonine protein kinase (STK, protein molecular weight about 125 kD), MAP kinase kinase kinase Ste11 (Ste11, protein molecular weight about 100 kD), pyruvate decarboxylase PdcA (PdcA, protein molecular weight about 65 kD), and phosphotransmitter protein Ypd1 (Ypd1, protein molecular weight about 20 kD). β-actin (about 43 kD) was used as control. To save on anti-non-phosphopeptide antibodies, PVDF membrane was cut based on molecular weight of different proteins. (c) The photographic films were performed using anti-phosphopeptide antibodies specific for ACC, STK, Ste11, PdcA and Ypd1. To save on anti-phosphopeptide antibodies, PVDF membrane was also cut based on molecular weight of

different proteins. The gel in a and the blots in b and c were run under the same experimental conditions.

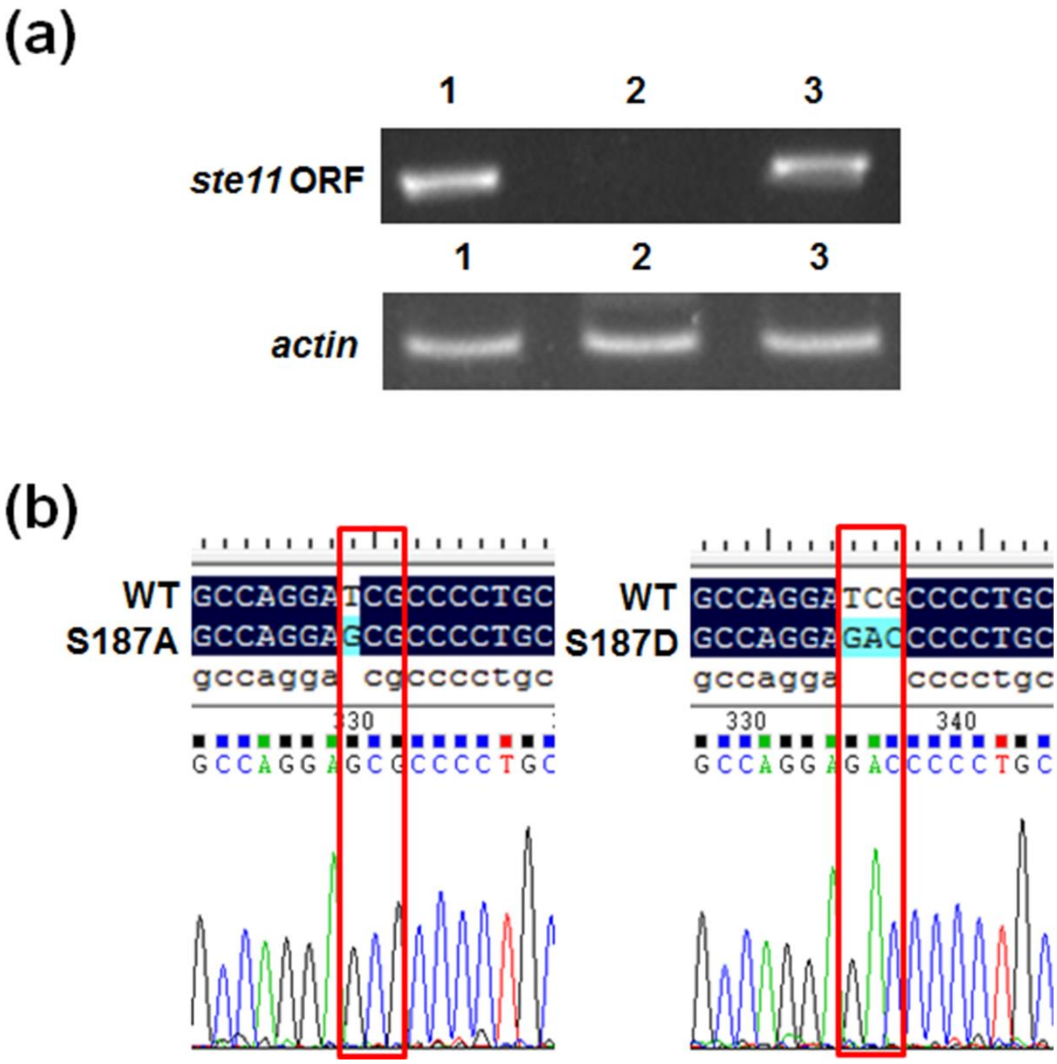

Supplementary Figure S4. Verification of WT and genetically engineered strains. (a) Open reading frame (ORF) verification of WT (Line 1), Δ*ste11* (Line 2) and COM (Line 3) strains with RT-PCR. Actin was used as a reference. (b) Sequence results of S187A and S187D mutants.

Supplementary Table S9. Primers used in this study.

Primer	Sequence5ˊ -3ˊ	Application
ste11-AF	TGCCGCTTTTACAAATCAGGAG	To amplify 5' region of *ste11*
ste11-AR	GGGTGAAGAGCATTGTTTGAGGCGCAACCTTAGAGTCTCGTCCCA	To amplify 5' region of *ste11*
ste11-BF	GCATCAGTGCCTCCTCTCAGACAACCAGCCTGGTTGGATGTG	To amplify 3' region of *ste11*
ste11-BR	TGTCAAGGCGGAGCTTCGTG	To amplify 3' region of *ste11*
ste11-AF1	AACTAAGCAGATACTGGTGCGTGT	fusion PCR of *ste11*
ste11-BR1	AGGATCAAGGACTGGGAGGGTGTC	fusion PCR of *ste11*
ste11-OF	GTTTCCAAGTTTAGCCTTCCTC	ORF verification
ste11-OR	CCAAATGTCTGCCTTCTTAGTG	ORF verification
pyrG-F	GCCTCAAACAATGCTCTTCACCC	To amplify *pyrG*
pyrG-R	GTCTGAGAGGAGGCACTGATGC	To amplify *pyrG*
P801-R	CAGGAGTTCTCGGGTTGTCG	overlap verification
P1020-F	ATCGGCAATACCGTCCAGAAGC	overlap verification
ste11-C-AF	CTATGACCATGATTACGCCAAGCTTTATGAATACCTTGCCTGCCTAT	To amplify complementation of *ste11*
ste11-C-AR	CCAGTGAATTCGAGCTCGGTACCTTCGTGGATTGAGAACACTTTA	To amplify complementation of *ste11*
S187A-AF	TCACGGCCAGGAGCGCCCCTGCGGGCCCAACGTTATGTTG	To amplify S187A
S187A-AR	CAACATAACGTTGGGCCCGCAGGGGCGCTCCTGGCCGTGA	To amplify S187A
S187D-AF	TCACGGCCAGGAGACCCCCTGCGGGCCCA	To amplify S187D

	ACGTTATGTTG	
S187D-AR	CAACATAACGTTGGGCCCGCAGGGGGTCTC CTGGCCGTGA	To amplify S187D
actin-F	CAGCCGCTAAGAGTTCCAG	To amplify *actin*
actin-R	CACCGATCCAAACCGAGTAC	To amplify *actin*
